# Therapeutic Effects of Nanocoating of Apitoxin (Bee Venom) and Polyvinyl Alcohol Supplemented with Zinc Oxide Nanoparticles

**DOI:** 10.3390/pharmaceutics17020172

**Published:** 2025-01-28

**Authors:** Husam Qanash, Abdulrahman S. Bazaid, Shahad F. Alharbi, Naif K. Binsaleh, Heba Barnawi, Bandar Alharbi, Ahmed Alsolami, Majed N. Almashjary

**Affiliations:** 1 Department of Medical Laboratory Science, College of Applied Medical Sciences, University of Ha’il, Hail 55476, Saudi Arabia; ar.bazaid@uoh.edu.sa (A.S.B.); alharbi.sh.f@gmail.com (S.F.A.); n.binsaleh@uoh.edu.sa (N.K.B.); h.barnawi@uoh.edu.sa (H.B.); b.alharbi@uoh.edu.sa (B.A.); 2 Medical and Diagnostic Research Center, University of Ha’il, Hail 55473, Saudi Arabia; 3 Department of Internal Medicine, College of Medicine, University of Ha’il, Hail 55476, Saudi Arabia; a.alsolami@uoh.edu.sa; 4 Department of Medical Laboratory Sciences, Faculty of Applied Medical Sciences, King Abdulaziz University, Jeddah 22254, Saudi Arabia; malmashjary@kau.edu.sa; 5 Hematology Research Unit, King Fahd Medical Research Center, King Abdulaziz University, Jeddah 22254, Saudi Arabia

**Keywords:** bee venom, therapeutic activity, polyvinyl alcohol, nanofilm, zinc oxide

## Abstract

**Background/Objectives:** Bee venom (BV), as a natural product, is one of the foundations of the pharmaceutical industry, through which many diseases, including serious ones, can be effectively treated. The BV nanofilm is an effective antidote delivered into the human body to target the affected area and address the issue without major side effects. In this study, we investigated the intriguing therapeutic effects of apitoxin (bee venom) used in isolation, combined with the powerful properties of zinc oxide nanoparticles. **Methods and Results:** BV nanofilm was evaluated using Fourier transform infrared (FTIR) spectroscopy and X-ray diffraction (XRD). The BV extract was analyzed using mass spectrometry (MS), which identified 84 active components present at varying concentrations. BV was treated with both polyvinyl alcohol (PVA) and zinc oxide nanoparticles (ZNPs) to increase the intensity of OH and CH_2_ groups and to enhance the dispersion of C=O. BV has demonstrated anti-type 2 diabetes activity by inhibiting α-amylase and α-glucosidase, which are starch-degrading enzymes. The nanofilm is an active mixture of BV, PVA, and ZNPs, which exhibited the highest antidiabetic activity with IC_50_ values of 30.33 μg/mL and 5.55 μg/mL for the inhibition of α-amylase and α-glucosidase, compared to IC_50_ of 51.69 µg/mL and IC_50_ of 7.30 µg/mL for BV, respectively. The nanofilm also showed higher anti-inflammatory activity by inhibiting red blood cell (RBC) hemolysis, with an IC_50_ of 16.99 μg/mL in comparison to IC_50_ of 72.99 µg/mL for BV alone. The nanofilm demonstrated broad-spectrum antimicrobial activity, effectively targeting both Gram-positive (*Staphylococcus aureus* ATCC 6538 and *Bacillus subtilis* ATCC 6633) and Gram-negative bacteria (*Salmonella typhi* ATCC 6539, *Escherichia coli* ATCC 8739). Furthermore, increased antioxidant activity was recorded by inhibiting the 1,1-diphenyl-2-picrylhydrazyl (DPPH) scavenging effect with an IC_50_ of 4.26 μg/mL and 19.43 μg/mL for nanofilm and BV, respectively. BV was found to be more toxic to liver tissue (HepG2 cell line) than nanofilm, with IC_50_ values of 18.5 ± 0.08 μg/mL and 52.27 ± 0.7 μg/mL, respectively. The BV extract displayed higher toxicity to liver tissue (2.3%) with 97.7% viability at 250 μg/mL, compared to nanofilm, which showed 0.09% toxicity and 99.9% viability at the same concentration. **Conclusions:** the BV nanofilm emerges as a promising alternative medicine, offering an innovative solution for treating various diseases through its high concentration of therapeutically active compounds and effortless targeting delivery.

## 1. Introduction

Drug therapy has achieved great success, especially in the last century following the development of biotechnology. However, drug therapy has side effects, some of which are severe, and some are common, including allergies to active ingredients. Therefore, it is necessary to seek alternative medicine to avoid severe side effects and provide highly effective treatment for diseases. Bee products, particularly bee venom (BV), are effective and safe natural medicines, capable of curing many diseases completely without side effects.

Apitherapy is one of the natural and effective approaches used since ancient times. Bee venom (BV) is a natural substance produced by bees, commonly used in the treatment of various diseases, particularly for its wound-healing properties [1]. A wide range of diseases can be treated with BV due to its various therapeutically active compounds, including biogenic amines, phospholipase A2, essential peptides (melittin, apamin, adolapin, mast cell-degranulating peptides), and water-soluble nitrogen-containing substances [2]. Despite being a small peptide composed of 26 amino acids, melittin is the predominant component of BV [3]. BV can be administered in different ways depending on the condition being treated, such as in the form of a cream, ointment, injection, acupuncture, or direct bee sting [4]. However, BV acupuncture is the most common method, wherein acupuncture points are directly injected with diluted BV. Therefore, this method is often used as an alternative treatment for patients suffering from pain and inflammatory diseases, such as rheumatoid arthritis, osteoarthritis, and neuritis [5,6]. In a broad sense, BV is considered a magic drug due to its multiple therapeutic effects in a short time, including antibacterial, antiviral, and anti-inflammatory activities. Furthermore, BV exhibits antiproliferative activity against cancer cells in various human organs [6]. BV is a mixture of water (88%), glucose, fructose, phospholipids, and proteins, with an acidic pH of 4.5 to 5.5. This mixture is a transparent, odorless liquid with a bitter taste and can dry easily, even at room temperature. Although BV is a transparent liquid, it can oxidize in air to form grayish-white crystals and may appear yellow under light [7].

Nanotechnology is a promising new approach used in many fields, including the pharmaceutical industry. Nanoparticles (NPs) of active ingredients are more effective and are delivered to the target site using nanoparticle-based drug delivery systems (DDSs). NPs must be stabilized by adding a strong, nonionic stabilizer, such as polyvinyl alcohol (PVA), which provides stable colloidal dispersion through stereostatic stabilization [8]. Although PVA is a water-soluble polyhydroxylated polymer that is easy to prepare, chemically stable, and biodegradable, its industrial use is limited by poor mechanical properties and thermal stability. Therefore, the mechanical properties and processing of PVA should be improved prior to its use in nanotechnology [9]. PVA is one of the most widely used stabilizers, promoting the production of small, stable molecules with a narrow size distribution. PVA has residual stability, which allows it to remain attached to the surface of polymer NPs even after repeated washing, forming a stabilizing layer that improves their resistance to freezing without the addition of a freeze protectant [10].

Zinc oxide nanoparticles (ZNPs) are widely used in nanotechnology due to their high optical, magnetic, and electrical efficiency. ZNPs are utilized in the manufacture of many products, including disinfectants for their antibacterial activity, as well as in anti-UV applications, deodorants, pharmaceuticals, electronics, gas sensors, and more [11,12,13]. However, ZNPs have negative effects on mammals with daily use, including genotoxic effects in the pancreas, stomach, liver, spleen, and thymus [14]; abnormalities in spermatogenesis; testicular damage; and decreased serum testosterone levels. Furthermore, ZNPs increase cytokine levels in the serum, leading to severe inflammation [15]. Fortunately, BV has an inhibitory effect on ZNPs that cause neuropathy and neurological abnormalities. The beneficial effects of BV are attributed to its ability to modulate neurotransmitter levels, maintain brain zinc concentration, enhance antioxidant activity, and reduce the expression of GAP-43 and neurofilaments in brain tissue [16]. ZNPs are frequently utilized in the production of various products, particularly in the medical and pharmaceutical sectors, owing to their exceptional properties, such as biocompatibility, UV protection, antimicrobial activity, enhanced thermal conductivity, high bonding energy, and elevated refractive index [17]. Metal oxide NPs are promising biomaterials with low toxicity and low accumulation. ZNPs are bioactive NPs with a unique safety profile and dermatological therapeutic properties as well [18,19]. This study aims to reveal the therapeutic potential of bee venom (BV) as a natural product without side effects, as BV acts as a broad-spectrum antibiotic, anti-inflammatory, antioxidant, and antidiabetic, all without causing critical toxicity to liver cells, which are responsible for absorbing toxins from the body and excreting them through feces.

## 2. Materials and Methods

### 2.1. Materials Used in the Study

A high sterile apitoxin (bee venom) was collected from a beekeeper, Mr. Mohammad A. Alaamri, Hail, Saudi Arabia. PVA (SIGMA-Aldrich Chemie GmbH, Taufkirchen, Germany, MW 146,000–186,000, 99+% hydrolyzed) and zinc acetate dihydrate (LOBA Chemie Pvt Ltd., Mumbai, India) were obtained, prepared, and stored according to the package instructions.

### 2.2. Collection, Storage, and Utilization of Bee Venom

The electric bee venom collection device, designed for in-hive use, comprises a device equipped with closely spaced wires connected to an electric current supplied by lithium batteries. To ensure the purity and quality of the collected bee venom for human use, a glass plate covered with food-grade shrink wrap is placed between the wires. The device is strategically positioned in crowded hives during periods of abundant foraging rich in pollen and nectar. This timing ensures that the collected venom adheres to standard specifications, specifically containing 50% of its dry weight in melittin. The electric bee venom device remains in the hive for a duration of 8–12 h. After this period, the device is carefully removed and transferred to a cool, shaded environment. To prevent contamination, beekeepers wear masks and gloves during the collection process. The food-grade shrink wrap covering the glass plate is removed, and the dry venom located in the center of the plate is meticulously scraped off using a sterilized scalpel. Venom along the edges of the glass plate, often contaminated with propolis and pollen grains, is discarded. The collected dry venom is stored in glass containers and placed in a freezer, where it maintains a shelf life of no less than two years. This harvested venom is subsequently utilized to produce various products, including creams, sublingual drops, and injection ampoules.

### 2.3. Formulation of Bioactive Film

A PVA solution (2% *w*/*v*) was prepared in a clean glass container by dissolving 2 g of PVA in 100 mL of distilled water. The PVA solution was supplemented with 0.1 g of BV while stirring for 3 h at 37 °C, resulting in a solution named PVA/BV. Zinc acetate dihydrate (0.01 g) was dissolved in 10 mL of distilled water and then added to 20 mL of the PVA/BV solution, with the mixture stirred overnight at 37 °C. The solution was pooled and subjected to ultrasonication using an ultrasonic probe for 5 min, followed by several washes with ethanol; this solution was named nanofilm. The PVA, PVA/BV, and nanofilm solutions were poured into 10 mm Teflon dishes and dried in a vacuum oven at 40 °C for 5 h [18].

### 2.4. Characterization Techniques

In the current study, several techniques were employed to evaluate the nanofilm. X-ray diffraction (XRD) patterns of the as-prepared compounds were obtained using an Empyrean PANalytical X-ray diffractometer [λ = 1.5418 Å (Bruker Nederland BV, Leiderdorp, The Netherlands)] with filtered Cu Kα radiation (45 kV and 30 mA). The diffraction angle (2θ) was scanned in the range of 10° to 80°, with a step size of 0.026° and a counting time of 20 s per step [20]. Furthermore, Fourier transform infrared spectroscopy (FTIR) spectra were recorded using a high-resolution FTIR spectrometer (JASCO FT/IR 460 Plus Spectrometer, Hachioji, Japan) [21]. Additionally, transmission electron microscopy (TEM) was conducted using a TEM-1230 (JEOL Corporation, Akishima, Tokyo, Japan) at an accelerating voltage of 100 kV [22]. Finally, scanning electron microscopy (SEM) (JSM-T20 JEOL, Chiyoda, Japan) was used to assess the morphology of the composites. The composite samples were mounted on a standard sample tray and coated with a thin layer of gold through deposition [23].

### 2.5. Mechanical Properties

The tensile strength (σR) and elongation at break (εR) were recorded using a Zwick electronic tensile testing machine (Zwick/Roell, Model Z010, Ulm, Germany). The hardness (Hv) was measured with a Shimadzu standard Vickers hardness tester (Kyoto, Japan), HP M-2 E precision hardness tester (344-04109-22) (Bruker Japan, Yokohama, Japan), applying a load of 1.96 N for 15 s on a sample with a thickness of approximately 1.5 mm [24].

### 2.6. In Vitro Assay of α-Amylase Inhibitory Activity

Alpha-amylase inhibitory activity was evaluated in vitro using the 3,5-dinitrosalicylic acid (DNSA) method [25]. The extract was first dissolved in a small amount of 10% DMSO and then further diluted in a buffer solution (Na_2_HPO_4_ 0.02 M, NaCl 0.006 M at pH 6.9) to achieve a concentration range of 1.9 to 1000 μg/mL. The extract (200 µL) was mixed with α-amylase solution (200 μL; 2 U/mL) and incubated for 10 min at 30 °C. After incubation, 200 μL of starch solution was added to each tube and incubated for an additional 3 min. Then, 200 μL of DNSA (containing 12 g of sodium potassium tartrate tetrahydrate, 8.0 mL of 2 M NaOH, and 20 mL of 96 mM 3,5-dinitrosalicylic acid solution) was added, and the mixture was boiled for 10 min in a water bath at 85–90 °C to stop the reaction. The mixture was allowed to cool to room temperature and then diluted with 5 mL of distilled water. The absorbance of the mixture was measured at 540 nm using a Biosystem 310 UV–Vis spectrophotometer (BIOBASE LLC, Jinan, China). A blank was prepared using only the plant extract without the enzyme solution. Alpha-amylase inhibitory activity was calculated using the following equation. The percentage of α-amylase inhibition was plotted against the concentration of the extract, and the IC_50_ value (the concentration required to inhibit 50% of the enzyme activity) was determined from the graph.

$$Alpha\;amylase\;inhibition\;(\%)=\frac{Abs.100\%\;\left(control\right)\;-\;Abs.sample}{Abs.100\%\;(control)}\times 100$$

### 2.7. In Vitro Assay of α-Glucosidase Inhibitory Activity

The extract was tested for α-glucosidase inhibitory activity using a modified method based on the protocol by Pistia and Hollingsworth [26]. Different concentrations (1.97 to 1000 μg/mL) of the extract (50 μL) were incubated with 10 μL of a 1 U/mL α-glucosidase solution (SIGMA EMD Millipore Corporation, Burlington, MA, USA) for 20 min at 37 °C, along with 125 μL of a 0.1 M phosphate buffer solution (pH 6.8). After incubation, 20 μL of 1 M p-nitrophenyl-α-D-glucopyranoside (pNPG) was added to initiate the reaction, and the mixture was incubated for an additional 30 min. The reaction was terminated by adding 50 μL of 0.1 N Na_2_CO_3_, and the absorbance was measured at 540 nm using a Biosystem 310 UV–Vis spectrophotometer. A blank was prepared using only the plant extract without the enzyme solution. Alpha-glucosidase inhibitory activity was calculated using the following equation. The percentage of α-glucosidase inhibition was plotted against the concentration of the extract, and IC_50_ values (the concentration required to inhibit 50% of the enzyme activity) were derived from the graph. One unit of α-glucosidase is defined as the amount of enzyme required to release one μmol of product (p-nitrophenol) from the substrate (pNPG) per minute.

$$Alpha\;glucosidase\;inhibition\;(\%)\;=\;\frac{100\;-\;(Abs.blank\;-\;Abs.sample)}{Abs.blank}\times 100$$

### 2.8. In Vitro Assay of Anti-Inflammatory Activity

Fresh, healthy blood (3 mL) was collected into heparinized tubes and centrifuged at 3000× *g* for 10 min. The erythrocyte granules were lysed using an equal volume of saline to the supernatant. The erythrocyte sediment was measured and reconstituted into a 40% *v/v* suspension with an isotonic buffer (10 mM sodium phosphate buffer, 0.2 g NaH_2_PO_4_, 1.15 g Na_2_HPO_4_, 9 g NaCl, pH 7.4 in 1.0 L distilled water). The reconstituted erythrocytes (resuspended supernatant) were then used for further experiments [27]. Different concentrations of the extract (100, 200, 400, 600, 800, 1000 μg/mL) were dissolved in 5 mL of distilled water to create hypotonic solutions in two pairs of centrifuge tubes (for each concentration). Isotonic solutions (5 mL) containing varying concentrations of the extract (3.9–1000 μg/mL) were prepared in two pairs of centrifuge tubes (for each concentration). The control tube contained 5 mL of distilled water and 5 mL of indomethacin. A 0.1 mL red blood cell suspension was added to each tube and mixed gently. The mixtures were incubated for 1 h at room temperature and then centrifuged for 3 min at 1300× *g*. The absorbance of the hemoglobin-containing supernatant was measured at 540 nm using a UV–visible spectrophotometer (Milton Roy, Houston, TX, USA). The hemolysis rate was calculated, assuming that hemolysis in the presence of distilled water was 100% [28]. The percentage of hemolysis inhibition using the extract was calculated using the following equation:

$$Inhibition\;of\;hemolysis\;(\%)=\frac{1-(Abs.2-Abs.1)}{Abs.3-Abs.1}\times 100$$

where Abs. 1 is the absorbance of the test sample in isotonic solution, Abs. 2 is the absorbance of the test sample in hypotonic solution, and Abs. 3 is the absorbance of the control sample in hypotonic solution.

### 2.9. Determination of Minimum Inhibitory Concentration (MIC)

The minimum inhibitory concentrations (MICs) of the tested extracts were determined using the microdilution method with Mueller–Hinton broth supplemented with lysed horse blood. The extracts were serially diluted twofold to achieve final concentrations ranging from 0.98 to 1000 μg/mL. Each dilution was distributed into the broth of individual wells. Fresh microbial cultures were prepared in a 0.85% sterilized NaCl solution to reach a final density of 3 × 10^6^ CFU/mL and were added to the wells. After 72 h of incubation at 35 °C under microaerobic conditions (15% CO_2_), the MIC was visually determined as the lowest extract concentration that completely inhibited the growth of the reference strain. Each microplate also included a positive control (inoculum only, no extract) and a negative control (extract only, no inoculum). Negative controls contained two serial dilutions (0.98–1000 μg/mL) of the test organism without inoculation to establish a baseline and avoid turbidity caused by the test material. A portion of the positive control was included to establish the initial concentration of the organism used. Turbidity was measured at 630 nm using a Biotech 800 TS microplate reader (Agilent, Lexington, MA, USA). The growth in wells containing antimicrobial agents was compared to that in growth control wells (not antimicrobial agents) and negative control wells. Growth endpoints were determined based on these comparisons. For the test to be valid, acceptable growth must be observed in the growth control wells.

### 2.10. Determination of Minimum Bactericidal Concentration (MBC)

The minimum bactericidal concentration (MBC) was determined by transferring 100 µL of microbial culture from each well showing complete inhibition of growth, as well as from the last positive control and the growth control, onto Mueller–Hinton agar plates supplemented with 10% sheep blood. The plates were incubated at 35 °C for 72 h under microaerophilic conditions. The MBC/MIC ratios were then calculated to assess whether the analyzed extracts exhibited a bactericidal or bacteriostatic effect [29].

### 2.11. In Vitro Assay of Antimicrobial Activity

The antimicrobial activity was determined in vitro using the agar well diffusion method [30]. Nutrient agar (for bacteria) or Sabouraud agar (for fungi) plates, seeded with the test microorganisms, were prepared and placed in the refrigerator for 1 h to reduce microbial growth. The agar surface was punctured to create several wells (6–8 mm) using a sterile cork borer. Each well was then inoculated with 100 μL of antimicrobial agents and test solutions. The inhibition zone around the well, indicating the inhibitory activity of the antimicrobial agent or test solution, was measured in millimeters, consistent with antimicrobial activity.

### 2.12. In Vitro Assay of Antioxidant Activity

The antioxidant (free radical scavenging) activity was assessed in vitro using the DPPH assay [31]. A 0.1 mM DPPH solution was prepared and mixed with ethanol, then 1 mL of this solution was added to 3 mL of the test sample at varying concentrations (3.9, 7.8, 15.62, 31.25, 62.5, 125, 250, 500, and 1000 μg/mL). The mixture was shaken thoroughly and allowed to stand at room temperature for 30 min. Absorbance was measured at 517 nm using a Milton Roy UV–Vis spectrophotometer. Ascorbic acid served as the standard, and the experiment was conducted in triplicate. The IC_50_ was determined from the log dose–inhibition curve. A lower absorbance of the reaction mixture reflected higher free radical scavenging activity. The percentage of DPPH scavenging was calculated using the following equation:

$$DPPH\;scavenging\;effect\;(\%)=\frac{Abs.control-Abs.sample}{Abs.control}\times 100$$

### 2.13. LC-ESI-MS/MS Analysis

Samples were analyzed using liquid chromatography–electrospray ionization–tandem mass spectrometry (LC-ESI-MS/MS) on a SCIEX Triple Quad 5500+ MS/MS system (SCIEX, Framingham, MA, USA), coupled with an ExionLC AC system (SCIEX, Framingham, MA, USA) for separation and an electrospray ionization (ESI) source for detection [32]. Separation was achieved on an Ascentis^®^ Express 90 Å C18 column (2.1 × 150 mm, 2.7 μm) (SIGMA EMD Millipore Corporation, Burlington, MA, USA). The mobile phase consisted of two solvents: (A) 5 mM ammonium formate (pH 3) and (B) acetonitrile (LC grade). The gradient program for the mobile phase was as follows: 0–1 min, 5% B; 5–20 min, 5% to 100% B; 20–25 min, 100% B; 25.01–30 min, back to 5% B. The flow rate was set at 0.3 mL/min, and the injection volume was 5 μL. For MS/MS analysis, negative ionization mode (EMS-IDA-EPI) was used for MS1 scans between 100 and 1000 Da with the following parameters: curtain gas at 25 psi, ion spray voltage at 5500 V, source temperature at 500 °C, and source gases 1 and 2 set to 45 psi. MS2 scans were performed from 50 to 1000 Da with a decoupling potential of 80 V and a collision energy of 35 eV.

### 2.14. Determination of Cytotoxicity and Viability of BV on the HepG2 Cell Line

A cell suspension (1 × 10^5^ cells/mL or 100 μL per well) was seeded into a 96-well tissue culture plate and incubated at 37 °C for 24 h to allow the formation of a complete monolayer. Once the monolayer was fully formed, the growth medium was removed, and the cells were washed twice with washing medium. The sample was then diluted twice in maintenance medium (RPMI) containing 2% serum, and 0.1 mL of each dilution was added to separate wells, leaving three wells as controls [33]. The plate was incubated at 37 °C for 24 h and subsequently examined for cytotoxicity, which was indicated by physical signs, such as partial or complete loss of the monolayer, as well as cell rounding, shrinkage, or degranulation [34]. A solution of MTT (3-4,5-dimethylthiazol-2-yl-2,5-diphenyltetrazolium bromide) (5 mg/mL in phosphate buffer) was prepared (BIO BASIC INC, Markham, ON, Canada). To each well of the tissue culture plate, 20 μL of the MTT solution was added and incubated at 37 °C with shaking at 150 rpm for 5 min to ensure thorough mixing with the medium. The plates were then incubated under the specified conditions (37 °C, 5% CO_2_) for 4 h, allowing the metabolites to fully develop. The medium was removed, and the plates were dried on paper towels to eliminate any residue. The metabolites in the MTT solution were then resuspended in 200 μL DMSO, stirred at 150 rpm for 5 min at 37 °C, and mixed completely. Absorbance was measured at 560 nm, with background subtraction at 620 nm [35,36]. Morphological changes can be observed on the cell surface or within the cytoskeleton of the cell lines. Damaged cells typically show a reduction in size due to significant loss of proteins and ions, resulting from altered permeability to sodium and potassium. Necrotic cells appear swollen, with chromatin clumping and the loss of the nuclear membrane. In contrast, apoptotic cells appear shrunken, with nuclear condensation followed by fragmentation of the nucleolus.

### 2.15. Statistical Analysis

The results (triads) are presented as mean ± standard deviation (SD) and mean ± standard error (SE), calculated using Microsoft Excel 365 and SPSS v.25. Quantitative data with a normal distribution across different treatments were analyzed using one-way analysis of variance (ANOVA) followed by Tukey’s post hoc test, with a significance level set at 0.05.

## 3. Results and Discussion

### 3.1. Structural and Interactional Insight: FTIR and XRD Characterization of Crystallinity in PVA, BV, and ZnO Nanofilm

**Figure 1:**
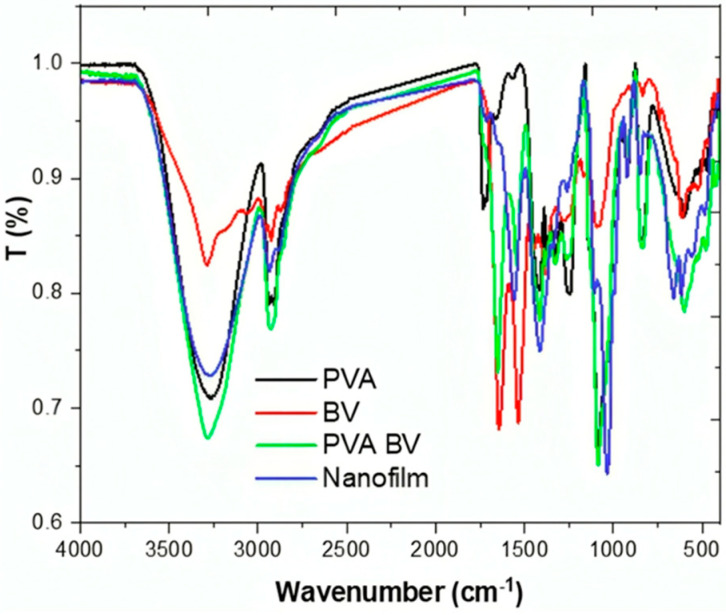
FTIR spectra of pure PVA, BV, PVA/BV, and nanofilm.

The nanofilm formulation was evaluated using physicochemical analysis, including FTIR and XRD. In the FTIR spectra, the PVA spectrum exhibited a typical pure PVA profile with peaks at 3260 cm^−1^ (OH stretching), 2939 cm^−1^ (CH_2_), 2905 cm^−1^ (CH_2_), 1735 cm^−1^ (C=O), 1555 cm^−1^ (C-H/O-H), and 1419 cm^−1^ (C-H/O-H) (Figure 1) [37]. The BV spectrum displayed bands at 3291 cm^−1^ that correspond to an overlap of N-H and O-H, 2930 cm^−1^ related to the stretching vibration of C-H, and 1640 and 1532 cm^−1^ assigned to amide I and II, respectively [38]. In this study, the FTIR spectrum of the PVA/BV blend showed increased intensity in the OH and CH_2_ bands, as well as a dispersion of C=O for PVA and amide I for BV. These changes affirm the interaction between PVA and BV. The addition of ZNPs affected the intensity of OH and CH_2_ groups, leading to a decrease, and the ZnO fingerprint bands were observed at 655 and 487 cm^−1^ in M-O and M-O-M, respectively [39]. Indeed, the FTIR spectra confirmed the successful formulation of the nanofilm.

**Figure 2:**
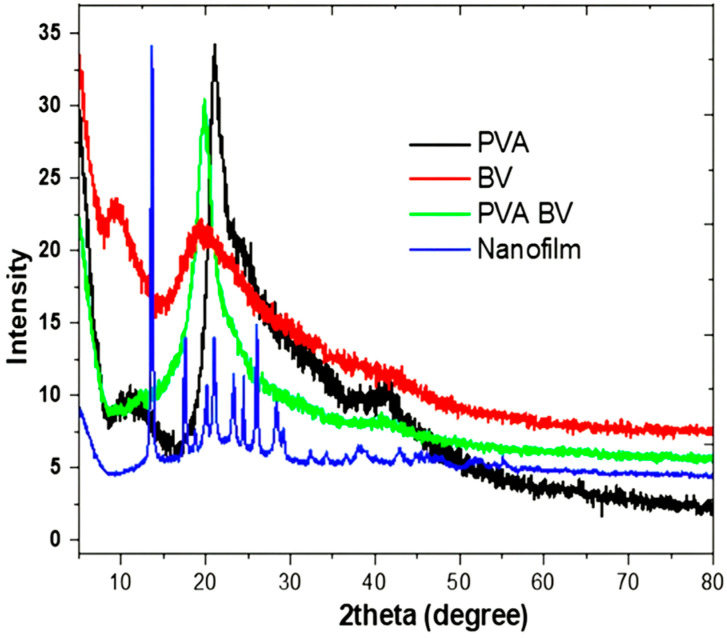
XRD pattern of pure PVA, BV, PVA/BV, and nanofilm.

The XRD pattern of pure PVA film revealed two characteristic peaks at 21° and 41°, which are associated with the semi-crystalline nature of PVA (Figure 2) [40]. Intra- and intermolecular hydrogen bonding in PVA supports its semi-crystalline structure. This type of bonding can occur within individual monomer units or between separate monomer units. The BV pattern exhibited amorphous behavior with two peaks at 9.1° and 19.27°, which is consistent with the literature [41]. Furthermore, the PVA/BV composition showed a pattern with two peaks at 1.9° and 41.2°, indicating the dominance of PVA crystallinity in the PVA/BV sample. Although ZNPs were added in small amounts (50 ppm), it was observed in the nanofilm pattern, which showed peaks at 32.5°, 34.4°, 36°, 46°, and 55°. All pronounced peaks can be indexed to the wurtzite zinc oxide structure (JCPDS data card no.: 36-1451) [42]. This study utilized the XRD technique to confirm the crystallization of the nanofilm and its pure materials, supporting the presence of ZNP doping in the film structure [43]. Moreover, the physicochemical analysis validated the molecular structure of the formulated nanoform.

### 3.2. Morphological Characterization of PVA, BV, and ZnO Nanofilm Through Scanning and Transmission Electron Microscopy

**Figure 3:**
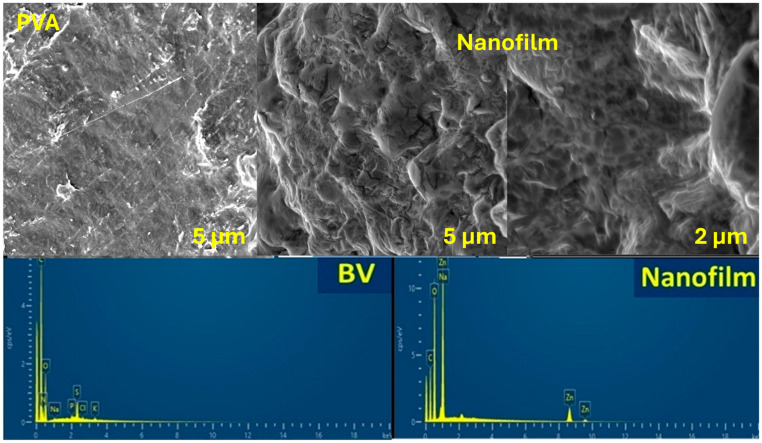
SEM images of pure PVA and nanofilm with low and high magnifications together with the EDX charts of BV and nanofilm.

After nanofilm formulation, the surface morphology was observed using FE-SEM of pure PVA (Figure 3). The PVA image showed a smooth surface of pure, nonsteroidal PVA [44]. However, the nanofilm formulation affected the surface morphology, which displayed numerous axes and depressions in the low magnification image. The high magnification image revealed a more porous surface due to BV integration, as well as some axes resembling coated spheres attached to the surface, indicating ZNPs that hindered the molecular structure of the nanofilm matrix. Furthermore, the EDX chart of BV presented elemental carbon, oxygen, nitrogen, phosphorus, sulfur, chloride, sodium, and potassium. These elements are related to the protein structure and other mineral elements. Additionally, the XRD pattern of the nanofilm indicated the removal of compositions, such as carbon, oxygen, sodium, and zinc. The peak oxygen density of the nanofilm was higher than that of pure PVA due to the formulation of ZNPs.

**Figure 4:**
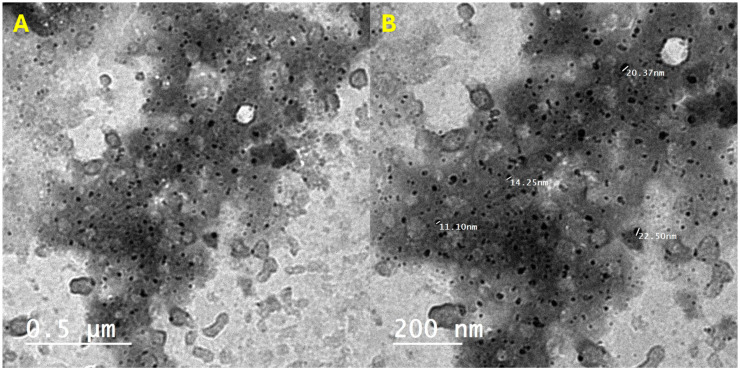
The HR-TEM images of nanofilm with (**A**) low (left) and (**B**) high (right) magnifications.

TEM images showed that both high and low magnification images displayed ZNPs as small irregular spheres with a size of approximately 17.1 nm (Figure 4). Specifically, TEM revealed a matrix consisting of at least two phases that could be PVA and BV, including dark particles that were homogeneously distributed ZNPs. These observations confirmed the formulation of nanostructured nanofilm with doped ZNPs of irregular size and homogeneous distribution. This nanofilm formulation holds promise as an alternative treatment in the pharmaceutical industry.

### 3.3. PVA, BV, and ZnO Nanofilm: A Potent Inhibitor of α-Amylase and α-Glucosidase for Enhanced Antidiabetic Efficacy

The breakdown of starch by starch-degrading enzymes, such as α-amylase and α-glucosidase, results in elevated blood sugar levels in diabetics. Consequently, inhibiting these enzymes serves as an antidiabetic strategy for type 2 diabetes. The same concentrations of acarbose, nanofilm, BV, PVA-blank (PVA-B), and PVA-BV were tested for their α-amylase inhibitory effects, with the mean absorbance measured at 540 nm. BV exhibited antidiabetic activity by inhibiting α-amylase, with an IC_50_ of 51.69 μg/mL, compared to the IC_50_ of acarbose, which served as a standard α-amylase inhibitor at 4.01 μg/mL. However, when BV was loaded onto PVA, it showed lower antidiabetic activity, inhibiting α-amylase with an IC_50_ of 182 μg/mL (Table 1). PVA-B displayed negligible inhibition of α-amylase, with no measurable IC_50_ value, indicating no antidiabetic activity. In contrast, nanofilm (BV, PVA, ZNPs) demonstrated the highest antidiabetic activity by inhibiting α-amylase with an IC_50_ of 30.33 μg/mL. Ahmad et al. reported that BV significantly reduced high blood sugar in diabetic mice, correlating with a notable increase in insulin levels [45]. Moreover, BV can significantly lower levels of malondialdehyde, cholesterol, triglycerides, creatinine, and aminotransferases compared to diabetic animals. Fortunately, BV has been found to enhance total antioxidant capacity, glutathione content, and catalase activity. Additionally, BV has shown potential in improving pancreatic function by enhancing the structure of the islets and regenerating β cells responsible for insulin secretion. Khulan et al. reported that BV could lower blood glucose levels while significantly increasing plasma insulin levels [46].

Alpha-glucosidase is comparable to α-amylase as an enzyme that hydrolyzes starch. However, α-amylase hydrolyzes the 1,4-glycosidic bonds in polysaccharides to release disaccharides, while α-glucosidase catalyzes the hydrolysis of disaccharides to monosaccharides, leading to postprandial hyperglycemia. Therefore, the inhibition of alpha-glucosidase activity is considered an antidote for type 2 diabetes. The same concentrations of acarbose, nanofilm, BV, PVA-B, and PVA-BV were evaluated for their α-glucosidase inhibitory activity, with the mean absorbance measured at 540 nm. BV exhibited antidiabetic activity by inhibiting α-glucosidase with an IC_50_ of 7.3 μg/mL, compared to the IC_50_ of acarbose as a standard α-amylase inhibitor, which is 2.57 μg/mL. However, when BV was loaded onto PVA, it showed lower antidiabetic activity, with an IC_50_ of 16.05 μg/mL (Table 2). PVA-B showed very low inhibition of α-glucosidase, with an IC_50_ of 736.78 μg/mL, indicating no significant antidiabetic activity. However, nanofilm exhibited the highest antidiabetic activity by inhibiting α-glucosidase, with an IC_50_ of 5.55 μg/mL. In the current study, the nanofilm of BV, PVA, and ZNPs effectively inhibits key starch-degrading enzymes (α-amylase and α-glucosidase), indicating a strong potential for managing type 2 diabetes. BV was administered in vivo for the management of diabetes, resulting in a reduction in serum glucose levels, followed by a significant increase in insulin levels compared to the diabetic rats [47]. Ryu et al. reported that α-glucosidase is a key enzyme responsible for the breakdown of maltose into glucose for absorption into the bloodstream in the small intestine [48]. Consequently, α-glucosidase inhibitors are considered an antidote for type 2 diabetes, as they help regulate the abnormal rise in plasma blood sugar after eating. Zhang et al. noted that although acarbose, voglibose, and miglitol are effective α-glucosidase inhibitors and approved antidotes for type 2 diabetes, they have adverse effects, such as abdominal cramping, flatulence, and diarrhea [49]. Therefore, natural products with therapeutic properties, such as bee products, are essential for use as nutraceuticals and antidiabetics. BV and propolis are versatile natural therapeutic products that exhibit various therapeutic activities, including antioxidant, anti-inflammatory, anticancer, antiviral, antibacterial, antifungal, and antidiabetic properties.

### 3.4. Augmented Anti-Inflammatory Activity of PVA-Treated Bee Venom: Mechanistic Insights into RBC Hemolysis Inhibition and DAMP Suppression

Red blood cell (RBC) breakdown results in the release of large amounts of inflammatory substances known as damage-associated molecular patterns (DAMPs), which enter the bloodstream and cause inflammation. Therefore, inhibiting RBC hemolysis prevents inflammation by stopping the release of DAMPs. Bee venom (BV) showed anti-inflammatory activity with an IC_50_ of 72.99 μg/mL, which decreased to 22.12 μg/mL with PVA-treated BV (PVA-BV), indicating higher anti-inflammatory activity of the latter compared to standard indomethacin, which has an IC_50_ of 5.86 μg/mL (Table 3). PVA-B demonstrated minimal anti-inflammatory activity, with an IC_50_ of 41.27 μg/mL. In contrast, nanofilm exhibited the highest anti-inflammatory activity, with an IC_50_ of 16.99 μg/mL.

**Figure 5:**
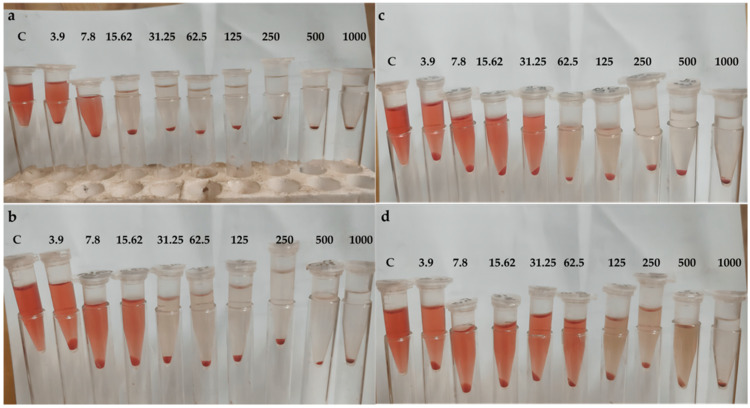
Inhibition of RBCs hemolysis by (**a**) nanofilm, (**b**) PVA-BV, (**c**) PVA-B, (**d**) BV; C, control.

The same concentrations of indomethacin, BV, PVA-BV, PVA-B, and nanofilm were examined for their inhibition of RBC hemolysis, with mean absorbance measured at 540 nm. The nanofilm demonstrated the highest inhibition of RBC hemolysis (98.7%) at 1000 μg/mL (Figure 5a), compared to indomethacin (99.4%), followed by PVA-BV (95.1%) (Figure 5b), PVA-B (93.1%) (Figure 5c), and BV (88.2%) (Figure 5d). In the present study, nanofilm inhibited red blood cell hemolysis, showcasing its anti-inflammatory effects.

Bellik reported that BV exerts an anti-inflammatory response by decreasing the expression of COX-2, PLA2, TNF-α, IL-1, IL-6, and NO, as well as reducing intracellular calcium levels [50]. Although BV is a mixture of many substances, melittin, adolapin, and tertiapin are primarily responsible for its anti-inflammatory effects. Both melittin and adolapin inhibit prostaglandin synthesis, thereby reducing the activities of cyclooxygenase and lipoxygenase. Dadar et al. found that tertiapin exhibits anti-inflammatory activity by blocking potassium channels [51].

### 3.5. Potent Antimicrobial Efficacy of Bee Venom, PVA-BV, and Nanofilm Against Diverse Bacterial and Fungal Strains

**Figure 6:**
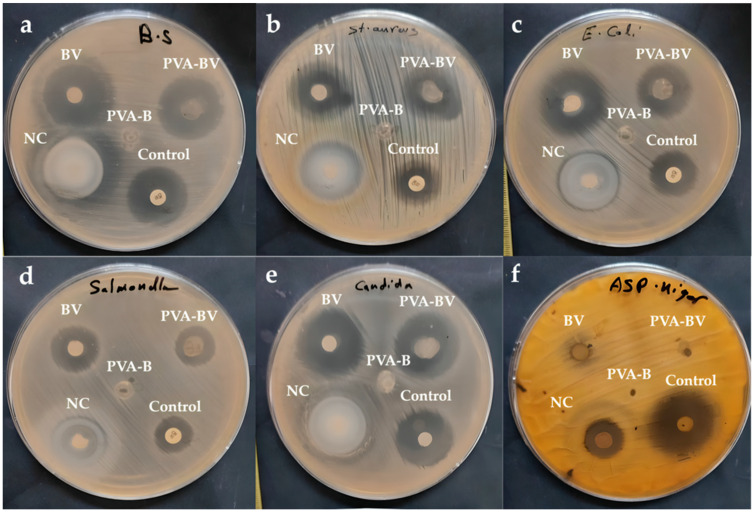
Antimicrobial activity against (**a**) *B. subtilis* ATCC 6633, (**b**) *S. aureus* ATCC 6538, (**c**) *E. coli* ATCC 8739, (**d**) *S. typhi* ATCC 6539, (**e**) *C. albicans* ATCC 10221, (**f**) *A. brasiliensis* ATCC 16888.

BV is an important active compound extracted from *Apis mellifera* L. (honeybee) that has antimicrobial activity, particularly against bacteria (both Gram-positive and Gram-negative) and fungi. Therefore, BV is considered a natural broad-spectrum antibiotic with minimal side effects. BV, PVA-BV, PVA-B, and nanofilm were tested for antibacterial activity in comparison to gentamicin (50 µg/mL) as a positive control, and for antifungal activity compared to fluconazole (32 µg/mL) as another positive control (Table 4). PVA-B did not demonstrate any antimicrobial activity, while nanofilm displayed the highest antimicrobial activity, except against *B. subtilis* ATCC 6633, where the nanofilm was found to have the same activity as PVA-BV and the control (Figure 6a). *S. aureus* ATCC 6538 was most susceptible to the nanofilm, followed by BV, PVA-BV, and control (Figure 6b). *E. coli* ATCC 8739 also showed the highest susceptibility to the nanofilm, followed by BV, PVA-BV, and the control (Figure 6c). *S. typhi* ATCC 6539 was most susceptible to the nanofilm; however, the effects of BV, PVA-BV, and the control were similar (Figure 6d). *C. albicans* ATCC 10221 exhibited the greatest susceptibility to the nanofilm and control, followed by BV and PVA-BV (Figure 6e). *A. brasiliensi* (formerly known as *A. niger*) ATCC 16888 was most susceptible to the control, followed by nanofilm, whereas BV and PVA-BV showed no activity (Figure 6f). This study showed that nanofilm exhibited broad-spectrum antibiotic activity against Gram-positive and Gram-negative bacteria, as well as molds like *Candida albicans* and *Aspergillus brasiliensis*. Haitham et al. reported that BV and its nanofilm (BV-V1, Carnica bee-V2, New Zealand type-V5) exhibited antibacterial activity against dental pathogens, including *Streptococcus* sp., *Enterococcus* sp., and *Bacillus* sp. at 50 µg/mL [52]. In addition to that, El-Sayied et al. (2024) reported that combining BV with nanoparticles significantly enhanced antibacterial efficacy against *Paenibacillus larvae* [53]. Barnes et al. documented that kanamycin, chloramphenicol, and streptomycin, as standard antibiotics, inhibited the growth of two *Streptococcus* isolates (*S. mutans* and *S. sanguinis*), while the five types of BV inhibited all *Streptococcus* isolates at concentrations of 30, 40, and 50 µg/mL [54]. Additionally, BV demonstrated antibacterial activity against *Enterococcus* sp. and *E. faecalis*. Hegazi et al. demonstrated that Gram-positive bacteria (both coagulase-positive and coagulase-negative *Staphylococcus*) were more sensitive to BV at lower concentrations than Gram-negative bacteria, such as *E. coli*, *Salmonella*, *E. cloacae*, and *Citrobacter freundii* [55].

The MIC and MBC in µg/mL were determined for BV, PVA-BV, and nanofilm against *B. subtilis* ATCC 6633, *S. aureus* ATCC 6538, *E. coli* ATCC 8739, *S. typhi* ATCC 6539, and *C. albicans* ATCC 10221 (Table 5). In *B. subtilis* ATCC 6633, the MICs of BV, PVA-BV, and nanofilm were similar, while the MBC of PVA-BV was higher than that of the homologous nanofilm and BV. In *S. aureus* ATCC 6538, the MIC of nanofilm was the lowest, followed by the MICs of BV and PVA-BV. The MBCs of nanofilm and BV were the same and higher than those of PVA-BV. In *E. coli* ATCC 8739, the MICs of nanofilm and BV were the same and lower than that of PVA-BV; similarly, the MBCs of nanofilm and BV were the same and lower than that of PVA-BV. In *S. typhi* ATCC 6539, the MIC of nanofilm was the lowest, followed by those of BV and PVA-BV, and the MBC of nanofilm was also the lowest, followed by BV and PVA-BV. In *C. albicans* ATCC 10221, the MIC of nanofilm was the lowest, followed by BV and PVA-BV, and the MBC of nanofilm was also the lowest, followed by BV and PVA-BV. Therefore, Gram-negative bacteria, especially *S. typhi* ATCC 6539, were more resistant to BV, PVA-BV, and nanofilm than Gram-positive bacteria and *C. albicans* ATCC 10221. Moreover, the nanofilm was a more effective antimicrobial agent than BV and PVA-BV. Haitham et al. [51] reported that the MICs of the nanofilm of BV were 31.31 µg/mL for *Enterococcus faecium*, 39.81 µg/mL for *Staphylococcus sciuri*, and 44.39 µg/mL for *Streptococcus mitis*, while the MBC for all three strains was almost the same (30 µg/mL). The MIC and MBC results demonstrated the efficacy of nanofilm against the tested microorganisms, with values of ≤31.25 µg/mL and 62.50 µg/mL, respectively.

### 3.6. Noteworthy Antioxidant Potency of Bee Venom (BV) and Nanofilm Demonstrated Through DPPH Radical Scavenging Assays

Antioxidant activity is assessed by the inhibition of lipid peroxidation, thus scavenging DPPH free radicals. The same concentrations of standard ascorbic acid, nanofilm, BV, PVA-B, and PVA-BV were tested for their DPPH scavenging effect, with the mean absorbance measured at 517 nm. BV showed antioxidant activity by scavenging DPPH with an IC_50_ of 19.43 μg/mL, compared to the IC_50_ of standard ascorbic acid, which is 2.35 μg/mL. PVA-BV exhibited higher antioxidant activity than BV, with an IC_50_ of 5.84 μg/mL (Table 6). PVA-B showed very low antioxidant activity that could be neglected, with an IC_50_ of 818.41 µg/mL. However, the nanofilm demonstrated the highest antioxidant activity, with an IC_50_ of 4.26 μg/mL. Somwongin et al. demonstrated that BV collected from European *A. mellifera* was found to have therapeutic effects, including antioxidant activity, due to its high capacity to scavenge DPPH [56]. Melittin, which is the largest component in the BV formulation (76.5 ± 1.9%), showed the highest inhibition of lipid peroxidation and DPPH removal. BV extracted from *A. mellifera*, especially melittin, demonstrated severe irritation (13.7 ± 0.5 at 2 mg/mL). Elswaby et al. reported that ethanolic extracts of BV samples collected from Kafr El-Sheikh, Fayoum, and Giza governorates were tested to determine that propolis was a more effective antioxidant than BV [57]. The antioxidant activities of BV and propolis extracted with ethanol, chloroform, and water were assessed using the DPPH method, with ascorbic acid as a control. The extracts exhibited different levels of free radical scavenging effect. More DPPH radicals were removed by BV than by propolis. Ethanolic extracts were found to be more effective in generating potent antioxidants. Ethanolic extracts of propolis collected from the Kafr El-Sheikh or Fayoum governorates at a concentration of 0.2 mg/mL demonstrated the highest free radical scavenging effects, achieving 94.0 ± 1.5% and 93.7 ± 1.8%, respectively, with no significant difference between the two regions. In contrast, the antioxidant properties of chloroform and water extracts of BV collected from the Giza governorate at identical concentrations were comparable and the lowest, showing 44.0 ± 1.5% and 43.0 ± 1.2%, respectively, with no significant difference between the two types of extracts. All extracts inhibited DPPH radical production in a dose-dependent manner, with efficacy increasing with concentration. At a high concentration (0.2 mg/mL), the antioxidant activities of the ethanolic extracts of propolis collected from Kafr El-Sheikh and Fayoum governorates were statistically like those of ascorbic acid, a powerful antioxidant used as a control. Rivero-Cruz et al. showed that the modern approach in the pharmaceutical industry involves using natural products, including bee products, especially BV and propolis, because of their therapeutic effects, including antioxidant properties [58]. The therapeutic effects of BV and propolis are attributed to several biologically active compounds, such as phenols and flavonoids in propolis, and melittin in BV. The antioxidants found in nanofilm and BV in this study will aid in the development of synthetic antioxidant compounds.

### 3.7. Promising Cytotoxicity of Bee Venom and Nanofilm Against HepG2 Cancer Cell Line

**Figure 7:**
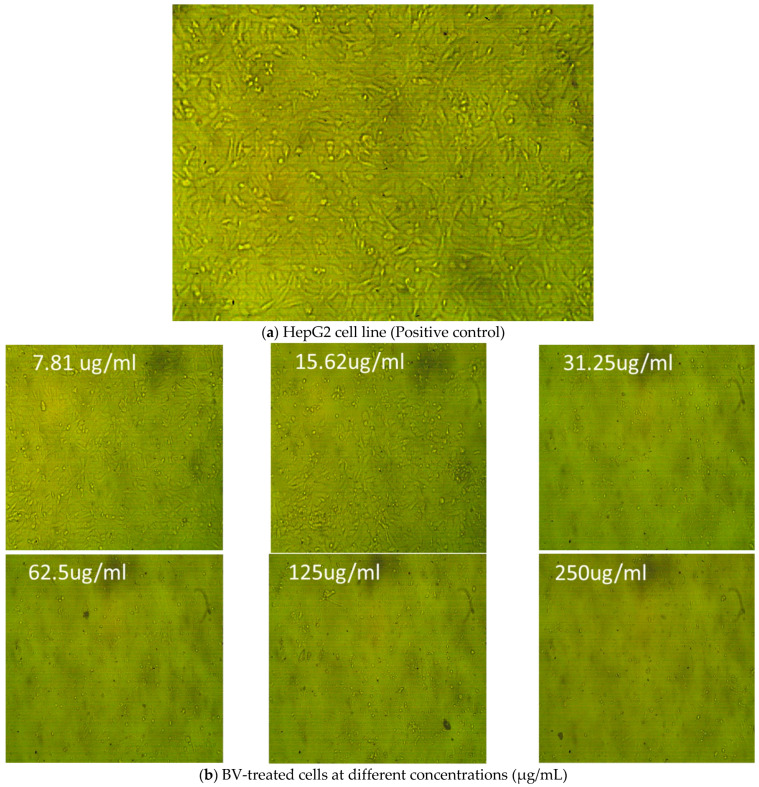

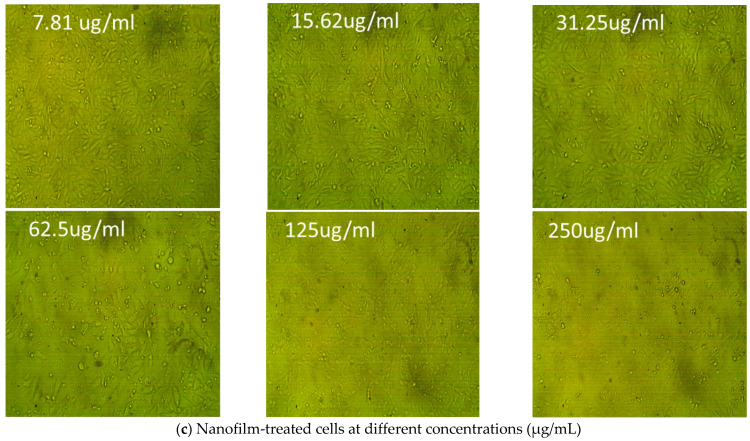
Cytotoxicity of BV and nanofilm on HepG2 cell line. (**a**) HepG2 cell line in liver tissues as a positive control, (**b**) different concentrations of BV, and (**c**) different concentrations of nanofilm for testing cytotoxicity.

**Figure 8:**
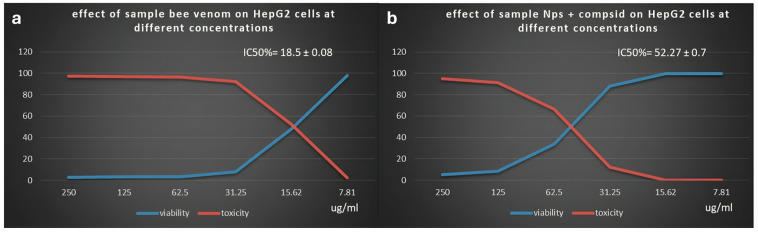
(**a**) Effect of BV on HepG2 cell line with an IC_50_ of 18.5 ± 0.08 µg/mL. (**b**) Effect of nanofilm on HepG2 cell line with an IC_50_ of 52.27 ± 0.7 µg/mL.

BV and nanofilm were tested for their effects (viability and toxicity) on the HepG2 cell line (liver tissue). The normal cell line did not show any abnormal manifestations, such as shrinkage or swelling (Figure 7a). Different concentrations of BV (Figure 7b) and nanofilm (Figure 7c) were tested for their effects on the cell line (Table 7). BV was more toxic to liver tissue (HepG2 cell line) than nanofilm, with an IC_50_ of 18.5 ± 0.08 μg/mL (Figure 8a) and 52.27 ± 0.7 μg/mL (Figure 8b), respectively. The BV extract showed higher toxicity to liver tissue (2.3%) with 97.7% viability at 250 μg/mL compared to the nanofilm, which showed 0.09% toxicity and 99.9% viability at the same concentration, demonstrating its potential for safer clinical applications. Although toxicity decreased and viability increased with decreasing concentrations of BV and nanofilm, the nanofilm was less toxic and more viable than BV. In comparison to our study, BV demonstrated an IC_50_ of 93.21 μg/mL against HepG2 cells [59]. Additionally, Kamel et al. (2024) reported that chitosan nanoparticles loaded with BV exhibited cytotoxicity against MCF-7 cells (IC_50_ of 437.2 μg/mL) and HepG2 cells (IC_50_ of 109.5 μg/mL) through the induction of ROS generation [60]. Furthermore, the anticancer potential of BV is attributed to its melittin content, which acts as an anticancer agent [61]. Young et al. reported that BV has various therapeutic activities, including anti-inflammatory, antimicrobial, antioxidant, and anticancer effects [62]. The toxicity of BV was investigated on normal human lymphocytes and HL-60 cells. Cells treated with BV at concentrations of 1 or 5 μg/mL were found to die within 24 h. However, when treated with BV at a concentration of 10 μg/mL, cell viability decreased over 72 h, potentially due to the half-life of BV. BV also completely inhibited the proliferation of these cells. Additionally, BV induced DNA fragmentation and micronuclei formation in HL-60 cells, and DNA fragmentation in human lymphocytes. The elevated levels of phosphatase and tensin homolog (PTEN) in HL-60 cells may arrest the cell cycle in the S phase. Upregulation of the forkhead transcription factors (FKHR and FKHRL1) in human lymphocytes after complete BV treatment may play a role in DNA damage repair and reducing genotoxicity. This suggests that BV may exert cytotoxic effects on these two cell types through different mechanisms. Finally, the anticancer experiment in the present study confirmed the potential of BV as an anticancer agent. However, further research is needed to evaluate the application of BV on a broader range of cancer cell lines to fully document and validate our results.

### 3.8. Comprehensive Profiling of Bioactive Compounds in Bee Venom via Mass Spectrometry (MS)

**Figure 9:**
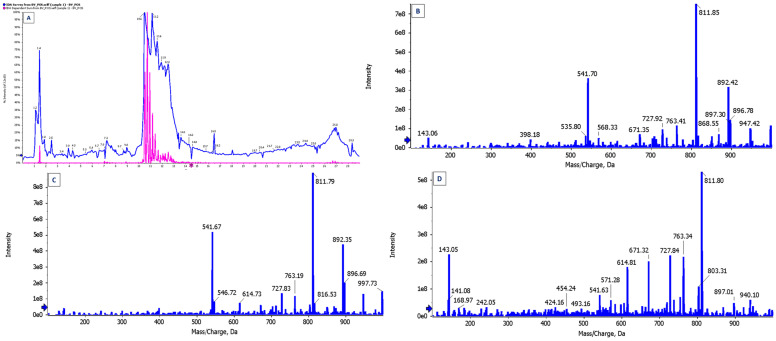
Microanalyses of BV components using (**A**) total in chromatogram (TIC); (**B**) mass spectrum with the highest intensity EPI (100–1000) from 10.934 min and precursor: 712.5 Da, CE: 35; (**C**) mass spectrum with the highest intensity EPI (100–1000) from 10.726 min and precursor: 712.2 Da, CE: 35; (**D**) mass spectrum with the highest intensity EPI (100–1000) from 10.520 min and precursor: 570.2 Da, CE: 35.

The BV extract was analyzed using MS as a specialized microanalysis technique to determine the type and concentration of its components. The BV extract was found to consist of 84 active components (Table 8) at various concentrations. The active components were identified by MS, where some were observed to repeat at different retention times, peak heights, and areas (Figure 9). Sharma and Rana found that the BV extract was analyzed into 41 individual compounds, which included hydrocarbons, alcohols, ketones, carboxylic acids, steroids, esters, retinoids, phenols, terpenes, amines, and others [63]. According to Jadhav et al. (2024), the liquid chromatography–mass spectrometry (LC-MS) analysis of BV nanoparticles revealed the presence of 23 bioactive compounds, among which melittin and apamin play significant roles in pharmacological applications [61]. The compounds with a retention time of 5.14—namely, α-Phellandrene, Bicyclo [3.1.0] Hexane, 4-methylene-1-(1-methylethyl)-, and α-Pinene—acquired the highest peak area, constituting 24.29% of the total peak area. Thangaleela et al. reported that terpenes, including phellandrene, pinene, terpinyl acetate, and andrographolide, recorded the highest peaks during the MS analysis of the BV extract and are known to possess biopharmacological activities [64]. Our analysis revealed several promising compounds that are suitable for a variety of pharmacological applications.

## 4. Conclusions

The growing trend toward sustainable treatments has driven significant advancements, particularly with the development of disposable films that enable continuous therapeutic release. In this study, a disposable, multifunctional, and biocompatible nanofilm was successfully developed, comprising PVA complexed with BV. The bee venom serves as both a stabilizer and a reducing agent, facilitating the conversion of bulk zinc to ZNPs. The structural properties of the nanofilm were characterized through physicochemical analyses, confirming the successful integration of PVA, BV, and ZNPs. Topographical investigations using FE-SEM and HR-TEM demonstrated a uniform distribution of ZNPs throughout the nanofilm. The nanofilm demonstrated effective inhibition of various pathogenic bacteria, with variable levels of activity. Furthermore, it displayed strong antioxidant properties (IC_50_ 4.26 μg/mL), surpassing BV (IC_50_ 19.43 μg/mL) in the DPPH assay. Interestingly, BV exhibited superior anticancer activity (IC_50_ 18.5 ± 0.08 μg/mL) compared to the nanofilm (IC_50_ 52.27 ± 0.7 μg/mL) against the HepG2 cell line. The BV-based nanofilm exhibited notable therapeutic potential, functioning as an antidote against hyperglycemia, inflammation, microbial proliferation, and oxidative stress induced by free radicals, all while showing no cytotoxic effects on liver cell lines. These findings highlight its potential as a next-generation biocompatible nanomaterial for managing chronic conditions, such as diabetes, inflammation, and microbial infections. The substantial content of therapeutically active compounds, combined with an efficient delivery mechanism, positions the BV nanofilm as a promising alternative therapeutic agent for a wide range of diseases.

## Figures and Tables

**Table 1:** In vitro assay of α-amylase inhibitory activity using standard acarbose, nanofilm, BV, PVA-B (blank), and PVA-BV.

S *	Factors	Concentrations (µg/mL)											IC_50_ (µg/mL)
		C	1.95	3.9	7.8	15.62	31.25	62.5	125	250	500	1000	
Acarbose	Mean A_540_	1.257	0.788	0.667	0.551	0.425	0.317	0.228	0.134	0.099	0.071	0.041	4.01
	E-inhibition (%)	0.000	37.30	47.00	56.10	66.20	74.80	81.90	89.30	92.20	94.40	96.80	
	SD *	0.002	0.003	0.005	0.003	0.002	0.003	0.002	0.002	0.003	0.004	0.003	
	SE *	0.000	0.001	0.001	0.001	0.001	0.001	0.001	0.001	0.001	0.001	0.001	
Nanofilm	Mean A_540_	1.257	1.208	0.937	0.826	0.708	0.594	0.484	0.375	0.257	0.160	0.098	30.33
	E-inhibition (%)	0.000	3.900	25.50	34.30	43.70	52.70	61.50	70.20	79.50	87.20	92.20	
	SD *	0.002	0.279	0.003	0.003	0.005	0.003	0.002	0.003	0.005	0.003	0.005	
	SE *	0.000	0.091	0.001	0.001	0.001	0.001	0.001	0.001	0.001	0.001	0.002	
BV	Mean A_540_	1.257	1.200	1.084	0.958	0.840	0.715	0.577	0.466	0.349	0.233	0.134	51.69
	E-inhibition (%)	0.000	4.600	13.80	23.80	33.10	43.10	54.10	62.90	72.20	81.50	89.30	
	SD *	0.002	0.015	0.007	0.003	0.003	0.004	0.004	0.005	0.002	0.006	0.003	
	SE *	0.000	0.005	0.002	0.001	0.001	0.001	0.001	0.001	0.001	0.002	0.001	
PVA-B *	Mean A_540_	1.257	1.254	1.254	1.253	1.250	1.247	1.238	1.233	1.229	1.118	0.918	None
	E-inhibition (%)	0.000	0.300	0.200	0.300	0.600	0.800	1.500	1.900	2.200	11.10	27.00	
	SD *	0.002	0.006	0.001	0.004	0.004	0.005	0.003	0.002	0.005	0.014	0.004	
	SE *	0.000	0.002	0.000	0.001	0.001	0.001	0.001	0.000	0.002	0.005	0.001	
PVA-BV	Mean A_540_	1.257	1.248	1.233	1.202	1.100	0.970	0.856	0.652	0.554	0.443	0.323	182
	E-inhibition (%)	0.000	0.700	1.900	4.400	12.50	22.80	31.90	48.20	56.00	64.70	74.30	
	SD *	0.002	0.005	0.007	0.012	0.003	0.003	0.002	0.003	0.003	0.002	0.008	
	SE *	0.000	0.001	0.002	0.004	0.001	0.001	0.000	0.001	0.001	0.000		

* S, sample; B, blank; E, enzyme; C, control; SD, standard deviation; SE, standard error; A, absorbance.

**Table 2:** In vitro assay of α-glucosidase inhibitory activity using standard acarbose, nanofilm, BV, PVA-B (blank), and PVA-BV.

S	Factors	Concentrations (µg/mL)											IC_50_ (µg/mL)
		C	1.95	3.9	7.8	15.62	31.25	62.5	125	250	500	1000	
Acarbose	Mean A_540_	1.817	0.983	0.861	0.735	0.620	0.501	0.393	0.284	0.213	0.106	0.047	2.57
	E-inhibition (%)	0.000	45.90	52.60	59.60	65.90	72.40	78.40	84.40	88.30	94.20	97.40	
	SD	0.004	0.004	0.003	0.003	0.002	0.006	0.003	0.002	0.002	0.007	0.004	
	SE	0.001	0.001	0.001	0.001	0.001	0.002	0.001	0.001	0.001	0.002	0.001	
Nanofilm	Mean A_540_	1.817	1.106	0.967	0.846	0.729	0.605	0.489	0.378	0.258	0.125	0.065	5.55
	E-inhibition (%)	0.000	39.10	46.80	53.40	59.90	66.70	73.10	79.20	85.80	93.10	96.40	
	SD	0.004	0.001	0.004	0.010	0.003	0.003	0.003	0.007	0.007	0.016	0.004	
	SE	0.001	0.000	0.001	0.003	0.001	0.001	0.001	0.002	0.002	0.005	0.001	
BV	Mean A_540_	1.817	1.179	1.011	0.886	0.773	0.647	0.514	0.401	0.269	0.170	0.112	7.3
	E-inhibition (%)	0.000	35.10	44.40	51.20	57.50	64.40	71.70	77.90	85.20	90.60	93.90	
	SD	0.004	0.004	0.005	0.003	0.002	0.003	0.004	0.004	0.003	0.004	0.004	
	SE	0.001	0.001	0.001	0.001	0.001	0.001	0.001	0.001	0.001	0.001	0.001	
PVA-B	Mean A_540_	1.817	1.723	1.642	1.546	1.431	1.343	1.279	1.158	1.072	0.950	0.855	736.78
	E-inhibition (%)	0.000	5.200	9.600	14.90	21.20	26.10	29.60	36.30	41.00	47.70	52.90	
	SD	0.004	0.011	0.018	0.012	0.003	0.006	0.003	0.004	0.005	0.007	0.028	
	SE	0.001	0.004	0.006	0.004	0.001	0.002	0.001	0.001	0.001	0.002	0.009	
PVA-BV	Mean A_540_	1.817	1.279	1.158	1.017	0.911	0.778	0.675	0.562	0.461	0.295	0.231	16.05
	E-inhibition (%)	0.000	29.60	36.30	44.00	49.80	57.20	62.80	69.10	74.60	83.80	87.30	
	SD	0.004	0.003	0.004	0.003	0.004	0.004	0.003	0.005	0.002	0.105	0.004	
	SE	0.001	0.001	0.001	0.001	0.001	0.001	0.001	0.001	0.000	0.034	0.001	

S, sample; B, blank; E, enzyme; C, control; SD, standard deviation; SE, standard error; A, absorbance.

**Table 3:** In vitro assay of anti-inflammatory activity using standard indomethacin, nanofilm, BV, PVA-B (blank), and PVA-BV.

S	Factors	Concentrations (µg/mL)										IC_50_ (µg/mL)
		C	3.9	7.8	15.62	31.25	62.5	125	250	500	1000	
IND	Mean A_540_	0.963	0.563	0.445	0.377	0.319	0.215	0.140	0.085	0.056	0.023	5.86
	A_540_ (IS)	0.001	0.000	0.000	0.000	0.003	0.003	0.009	0.01	0.013	0.017	
	H-inhibition (%)	0.000	41.50	53.80	60.90	67.10	78.00	86.20	92.20	95.50	99.40	
	SD	0.013	0.006	0.005	0.003	0.005	0.002	0.003	0.005	0.002	0.007	
	SE	0.004	0.002	0.002	0.001	0.002	0.001	0.001	0.001	0.001	0.002	
Nanofilm	Mean A_540_	0.963	0.719	0.616	0.489	0.386	0.281	0.159	0.108	0.083	0.056	16.99
	A_540_ (IS)	0.001	0.000	0.001	0.005	0.008	0.014	0.018	0.023	0.032	0.044	
	H-inhibition (%)	0.000	25.40	36.00	49.50	60.40	71.80	85.00	91.00	94.60	98.70	
	SD	0.007	0.006	0.003	0.007	0.004	0.003	0.003	0.003	0.004	0.003	
	SE	0.002	0.002	0.001	0.002	0.001	0.001	0.001	0.001	0.001	0.001	
BV	Mean A_540_	0.963	0.887	0.793	0.702	0.611	0.499	0.417	0.324	0.244	0.152	72.99
	A_540_ (IS)	0.001	0.000	0.001	0.005	0.008	0.014	0.018	0.023	0.032	0.044	
	H-inhibition (%)	0.000	7.900	17.70	27.30	36.90	48.90	57.80	68.00	77.20	88.20	
	SD	0.007	0.063	0.002	0.003	0.005	0.002	0.004	0.006	0.003	0.004	
	SE	0.002	0.020	0.001	0.001	0.001	0.000	0.001	0.002	0.001	0.001	
PVA-B	Mean A_540_	0.963	0.820	0.730	0.647	0.530	0.418	0.316	0.205	0.134	0.107	41.27
	A_540_ (IS)	0.001	0.000	0.001	0.005	0.008	0.014	0.018	0.023	0.032	0.044	
	H-inhibition (%)	0.000	14.80	24.20	33.00	45.40	57.50	68.50	80.60	89.00	93.10	
	SD	0.007	0.004	0.002	0.004	0.003	0.004	0.003	0.004	0.002	0.003	
	SE	0.002	0.001	0.001	0.001	0.001	0.001	0.001	0.001	0.001	0.001	
PVA-BV	Mean A_540_	0.963	0.737	0.652	0.518	0.437	0.309	0.216	0.163	0.110	0.089	22.12
	A_540_ (IS)	0.001	0.000	0.001	0.005	0.008	0.014	0.018	0.023	0.032	0.044	
	H-inhibition (%)	0.000	23.40	32.30	46.50	55.10	68.90	79.10	85.10	91.60	95.10	
	SD	0.007	0.003	0.003	0.006	0.009	0.001	0.003	0.004	0.005	0.009	
	SE	0.002	0.001	0.001	0.002	0.003	0.000	0.001	0.001	0.002	0.003	

S, sample; IND, indomethacin; B, blank; H, hemolysis; IS, isotonic solution; C, control; SD, standard deviation; SE, standard error; A, absorbance.

**Table 4:** In vitro analysis of antimicrobial activity of nanofilm, BV, PVA-B (blank), and PVA-BV.

Taxon	Pathogen	Antimicrobial Activity (mm) of Sample				
		Control	BV	PVA-BV	PVA-B	Nanofilm
Gram-positive bacteria	*B. subtilis* ATCC 6633	23 ± 0.2	25 ± 0.1	23 ± 0.1	NA	23 ± 0.2
	*S. aureus* ATCC 6538	16 ± 0.2	21 ± 0.2	19 ± 0.2	NA	24 ± 0.2
Gram-negative bacteria	*E. coli* ATCC 8739	17 ± 0.1	24 ± 0.2	19 ± 0.2	NA	25 ± 0.1
	*S. typhi* ATCC 6539	14 ± 0.2	17 ± 0.1	14 ± 0.2	NA	21 ± 0.1
Molds	*C. albicans* ATCC 10221	23 ± 0.2	25 ± 0.2	22 ± 0.2	NA	32 ± 0.2
	*A. brasiliensis* ATCC 16888	25 ± 0.1	NA	NA	NA	15 ± 0.1

B, blank; NA, no activity. Gentamycin (50 µg/mL) is a control for bacteria; fluconazole (32 µg/mL) is a control for molds.

**Table 5:** Determination of MIC and MBC of nanofilm, BV, and PVA-BV.

Pathogen	MIC (µg/mL)			MBC (µg/mL)		
	BV	PVA-BV	Nanofilm	BV	PVA-BV	Nanofilm
*B. subtilis* ATCC 6633	15.62	15.62	15.62	15.62	31.25	15.62
*S. aureus* ATCC 6538	31.25	62.50	15.62	31.25	125.0	31.25
*E. coli* ATCC 8739	15.62	62.50	15.62	31.25	250.0	31.25
*S. typhi* ATCC 6539	125.0	250.0	31.25	250.0	500.0	62.50
*C. albicans* ATCC 10221	31.25	125.0	15.62	62.50	500.0	15.62

MIC, minimum inhibitory concentration; MBC, minimum bactericidal concentration.

**Table 6:** In vitro assay of antioxidant activity using standard ascorbic acid, nanofilm, BV, PVA-B (blank), and PVA-BV.

S	Factors	Concentrations (µg/mL)											IC_50_ (µg/mL)
		C	1.95	3.9	7.8	15.62	31.25	62.5	125	250	500	1000	
AA	Mean A_517_	1.591	0.882	0.734	0.640	0.519	0.393	0.252	0.136	0.094	0.058	0.030	2.35
	DPPH scav. (%)	0.000	44.60	53.90	59.80	67.40	75.30	84.20	91.50	94.10	96.30	98.10	
	SD	0.003	0.003	0.003	0.004	0.002	0.002	0.003	0.003	0.003	0.002	0.003	
	SE	0.001	0.001	0.001	0.001	0.000	0.000	0.001	0.001	0.001	0.001	0.001	
Nanofilm	Mean A_517_	1.591	0.946	0.827	0.695	0.587	0.502	0.391	0.285	0.181	0.106	0.085	4.26
	DPPH scav. (%)	0.000	40.50	48.00	56.30	63.10	68.50	75.40	82.10	88.60	93.30	94.70	
	SD	0.003	0.001	0.004	0.002	0.004	0.004	0.003	0.003	0.003	0.003	0.003	
	SE	0.001	0.000	0.001	0.001	0.001	0.001	0.001	0.001	0.001	0.001	0.001	
BV	Mean A_517_	1.591	1.183	1.062	0.942	0.831	0.706	0.595	0.492	0.373	0.261	0.158	19.43
	DPPH scav. (%)	0.000	25.60	33.20	40.80	47.70	55.60	62.60	69.10	76.60	83.60	90.10	
	SD	0.003	0.003	0.007	0.004	0.003	0.003	0.003	0.003	0.005	0.003	0.004	
	SE	0.001	0.001	0.002	0.001	0.001	0.001	0.001	0.001	0.001	0.001	0.001	
PVA-B	Mean A_517_	1.591	1.556	1.538	1.520	1.478	1.390	1.289	1.102	0.961	0.802	0.677	818.41
	DPPH scav. (%)	0.000	2.200	3.300	4.400	7.100	12.60	19.00	30.70	39.60	49.60	57.40	
	SD	0.003	0.005	0.005	0.007	0.007	0.007	0.004	0.006	0.006	0.004	0.004	
	SE	0.001	0.001	0.002	0.002	0.002	0.002	0.001	0.002	0.002	0.001	0.001	
PVA-BV	Mean A_517_	1.591	0.993	0.871	0.747	0.636	0.524	0.430	0.318	0.212	0.142	0.105	5.84
	DPPH scav. (%)	0.000	37.60	45.30	53.00	60.00	67.10	73.00	80.00	86.70	91.10	93.40	
	SD	0.003	0.004	0.004	0.003	0.006	0.006	0.007	0.004	0.004	0.003	0.003	
	SE	0.001	0.001	0.001	0.001	0.002	0.002	0.002	0.001	0.001	0.001	0.001	

S, sample; AA, ascorbic acid; B, blank; C, control; SD, standard deviation; SE, standard error; A, absorbance.

**Table 7:** Cytotoxicity of BV and nanofilm on HepG2 cell line.

Sample	Conc. (µg/mL)	Mean A_560_	Viability (%)	Toxicity (%)	SE	IC_50_ ± SD (µg/mL)
HepG2 cell line	None	0.722	100.0	0.000	0.002887	-----------
BV	250.0	0.020	2.816251154	97.18374885	0.001453	18.5 ± 0.08
	125.0	0.022	3.047091413	96.95290859	0.001528	18.5 ± 0.08
	62.50	0.025	3.462603878	96.53739612	0.001732	18.5 ± 0.08
	31.25	0.057	7.894736842	92.10526316	0.002082	18.5 ± 0.08
	15.62	0.343	47.59926131	52.40073869	0.004410	18.5 ± 0.08
	7.810	0.705	97.73776547	2.262234534	0.006692	18.5 ± 0.08
Nanofilm	250.0	0.037	5.12465374	94.87534626	0.003606	52.27 ± 0.7
	125.0	0.061	8.541089566	91.45891043	0.002028	52.27 ± 0.7
	62.50	0.241	33.47183749	66.52816251	0.006489	52.27 ± 0.7
	31.25	0.635	88.04247461	11.95752539	0.006333	52.27 ± 0.7
	15.62	0.720	99.81532779	0.184672207	0.001202	52.27 ± 0.7
	7.810	0.721	99.9076639	0.092336103	0.002028	52.27 ± 0.7

SD, standard deviation; SE, standard error.

**Table 8:** Components of BV based on microanalysis.

Compound	RT (min)	Height	Area	Compound	RT (min)	Height	Area
Phenylhydrazine	1.038317	3,359,283	4.69 × 10^7^	IAA	3.470867	948,240.3	2.02 × 10^7^
1,4-benzoquinone	1.100533	5,172,849	9.17 × 10^7^	MTE	3.56935	9,819,045	1.43 × 10^8^
Cytosine	1.100533	4.90 × 10^7^	6.78 × 10^8^	PCALS	3.56935	5,479,700	9.26 × 10^7^
1-methylhistamine	1.100533	1.16 × 10^7^	1.94 × 10^8^	Creatinine	3.645717	937,919.4	1.37 × 10^7^
L-aspartic acid	1.100533	1.06 × 10^7^	1.16 × 10^8^	PCALS	3.745333	6,324,281	2.04 × 10^8^
L-β-homothreonine	1.100533	1.06 × 10^7^	1.16 × 10^8^	Creatinine	3.92655	1,375,321	2.75 × 10^7^
L-β-homotyrosine-HCl	1.100533	6,995,533	9.28 × 10^7^	PCALS	4.105233	2,293,368	2.49 × 10^7^
NPP	1.100533	5,402,951	7.66 × 10^7^	DHGP	5.647417	2,531,331	5.33 × 10^7^
N,N-dimethylglycine	1.185783	4.83 × 10^7^	8.08 × 10^8^	*trans*-Cinnamate	5.9459	7,588,287	1.60 × 10^8^
Histamine	1.185783	4.01 × 10^7^	5.63 × 10^8^	Pyridoxine	6.401083	3,333,806	4.00 × 10^7^
Adenine	1.2593	2.75 × 10^7^	3.98 × 10^8^	1,4-Benzoquinone	6.730533	3,552,315	8.95 × 10^7^
Adenine	1.2593	2.75 × 10^7^	3.95 × 10^8^	Glycyrrhizate	6.960783	7,946,545	1.19 × 10^8^
HAA	1.2593	5.67 × 10^7^	1.18 × 10^9^	IBPPA	7.03055	2,657,344	4.33 × 10^7^
*cis*-aconitate	1.2593	1.71 × 10^7^	4.21 × 10^8^	PCALS	7.212983	3,458,333	4.78 × 10^7^
4-pyridoxate	1.2593	2.07 × 10^7^	3.19 × 10^8^	Dihydrocapsaicin	7.791934	1,730,243	1.79 × 10^7^
Histamine	1.328533	3.94 × 10^7^	5.94 × 10^8^	PDG	7.990833	7,315,209	8.00 × 10^7^
P-hydroxybenzoic acid	1.38935	2.92 × 10^7^	5.05 × 10^8^	Glycyrrhizate	8.073916	1,187,500	1.80 × 10^7^
Guanine	1.435283	1.91 × 10^7^	2.17 × 10^8^	DAMP	8.343884	813,496.5	1.57 × 10^7^
Phenaturic acid	1.435283	2,697,917	5.57 × 10^7^	Esculin	8.813416	2,678,536	2.40 × 10^7^
Carnosine	1.435283	2.13 × 10^7^	2.50 × 10^8^	UMP	9.382533	2,979,774	4.79 × 10^7^
Carnosine	1.435283	2.13 × 10^7^	1.96 × 10^8^	UMP	9.382533	2,979,774	5.11 × 10^7^
Hypoxanthine	1.4947	3.45 × 10^7^	4.94 × 10^8^	NADH	10.85075	6,150,434	5.09 × 10^7^
N-acetyl-L-Cysteine	1.4947	8,048,392	1.57 × 10^8^	GDM	12.23113	5,386,385	4.52 × 10^7^
L-methionine sulfone	1.4947	1.18 × 10^7^	1.34 × 10^8^	KRR	12.33495	3,699,226	3.30 × 10^7^
Tyr	1.4947	1.18 × 10^7^	1.34 × 10^8^	DGPSA	14.23322	1,514,209	1.28 × 10^7^
Histamine	1.572317	2.45 × 10^7^	6.97 × 10^8^	Dihydrosphingosine	15.22845	2,512,405	4.27 × 10^7^
Histamine	1.572317	2.45 × 10^7^	3.44 × 10^8^	Etidronate	18.47452	2,209,014	4.36 × 10^7^
N-isovaleroylglycine	1.572317	1.14 × 10^7^	1.32 × 10^8^	MHGP	19.03983	1,104,625	2.65 × 10^7^
HMPPE	1.572317	5,544,233	7.17 × 10^7^	MSPG	20.54685	1,843,891	4.36 × 10^7^
Uric acid	1.6829	5,990,202	7.77 × 10^7^	Creatinine	21.13187	5,952,579	1.73 × 10^8^
Cysteine S-sulfate	1.78405	1.59 × 10^7^	2.08 × 10^8^	Etidronate	21.6648	1,927,646	2.90 × 10^7^
DCA	1.78405	1.35 × 10^7^	1.78 × 10^8^	Xylitol	24.34558	6,596,503	1.66 × 10^8^
NPP	1.78405	5,439,595	7.60 × 10^7^	Isoguvacine	25.56753	1.21 × 10^7^	1.83 × 10^8^
Carnosine	1.78405	1.45 × 10^7^	2.25 × 10^8^	Isoguvacine	26.68405	1.14 × 10^7^	1.68 × 10^8^
PSA	1.862533	6,396,178	1.22 × 10^8^	Isoguvacine	27.34783	1.15 × 10^7^	2.11 × 10^8^
HICA	2.28075	4,375,156	6.77 × 10^7^	trans-Cinnamate	27.55965	3,016,423	4.99 × 10^7^
Quisqualate	2.28075	3,740,382	6.14 × 10^7^	Isoguvacine	27.78005	1.27 × 10^7^	3.12 × 10^8^
N-acetylglutamate	2.28075	3,740,557	6.05 × 10^7^	Isoguvacine	28.00437	1.20 × 10^7^	2.81 × 10^8^
PSA	2.28075	3,469,233	5.54 × 10^7^	Cerulenin	28.33712	3,365,315	5.61 × 10^7^
PCALS	3.1939	6,083,586	1.60 × 10^8^	Cerulenin	28.5104	3,407,139	7.71 × 10^7^
Phenylhydrazine	3.3769	4,312,904	7.04 × 10^7^	Etidronate	28.61048	2,563,221	6.73 × 10^7^
PCALS	3.3769	3,979,634	5.59 × 10^7^				

HMPPE, 3,4-hydroxy-3-methoxyphenyl-prop-2-enoic acid; DCA, 3,4-dimethoxycinnamic acid; NPP, 4-nitrophenyl phosphate; HAA, 3-hydroxyanthranilic acid; PSA, piperacillin sodium salt; HICA, 1H-indole-3-carboxylic acid; PCALS, n-propionyl coenzyme A lithium salt; IAA, β-indoleacetic acid; MTE, 4-methyl-5-thiazoleethanol; DHGP, 1-decanoyl-2-hydroxy-sn-glycero-3-phosphocholine; IBPPA, 2-(4-isobutylphenyl) propionic acid; PDG, 3-phospho-D-glycerate; DAMP, 2′-deoxyadenosine 5′-monophosphate; UMP, uridine 5′-monophosphate; NADH, nicotinamide adenine dinucleotide H; GDM, guanosine 5′-diphosphate-D-mannose; KRR, Kaempferol-3-O-robinoside-7-O-rhamnoside; DGPSA, 1,2-dipalmitoyl-sn-glycero-3-phospho-rac-(1-glycerol) sodium salt; MHGP, 1-myristoyl-2-hydroxy-sn-glycero-3-phosphate; MSPG, 3-(Methylsulfinyl) propylglucosinolate.

## Data Availability

Data that supports the findings of this study are available within the article and from the corresponding author upon request.
